# Atlanta Medical College Museum

**Published:** 1859-06

**Authors:** 


### ATLANTA MEDICAL COLLEGE MUSEUM.

We are indebted to Dr. Hugh Oglesby, a prominent physician of Madison, Georgia, for a valuable and interesting addition to the museum.

To those who are feeling an interest in the success of that department of the Institution, we would say, that it is already presenting (in the estimation of those who are qualified to judge, and not connected with the Institution,) a highly-respectable appearance, and furnishing large facilities for the acquisition of medical knowledge.

Under the management of the efficient Curator, Dr. N. D'Alvigny, it will constantly improve, and will, we hope, soon compare favorably in value, if not in extent, with the museums of the oldest college in the country.

We would still urge our friends to send in any thing of interest or value, that they may be disposed to contribute, or which, indeed, they would like to have preserved, which cannot be done with any certainty, except in a museum.

The expenses of transportation will, of course, be paid by the college. Address N. D'Alvigny, M. D., Curator of Atlanta Medical College, Atlanta, Ga.

